# Development and Validation of a Radiomics Nomogram Using Computed Tomography for Differentiating Immune Checkpoint Inhibitor-Related Pneumonitis From Radiation Pneumonitis for Patients With Non-Small Cell Lung Cancer

**DOI:** 10.3389/fimmu.2022.870842

**Published:** 2022-04-26

**Authors:** Qingtao Qiu, Ligang Xing, Yu Wang, Alei Feng, Qiang Wen

**Affiliations:** ^1^ Department of Radiation Physics and Technology, Shandong Cancer Hospital and Institute, Shandong First Medical University and Shandong Academy of Medical Sciences, Jinan, China; ^2^ Department of Radiation Oncology, Shandong Cancer Hospital and Institute, Shandong First Medical University and Shandong Academy of Medical Sciences, Jinan, China; ^3^ Department of Radiation Oncology, Shandong Provincial Hospital Affiliated to Shandong First Medical University, Shandong First Medical University, Jinan, China

**Keywords:** radiomics nomogram, immune checkpoint inhibitor-related pneumonitis, radiation pneumonitis, NSCLC, differential diagnosis

## Abstract

**Background:**

The combination of immunotherapy and chemoradiotherapy has become the standard therapeutic strategy for patients with unresected locally advance-stage non-small cell lung cancer (NSCLC) and induced treatment-related adverse effects, particularly immune checkpoint inhibitor-related pneumonitis (CIP) and radiation pneumonitis (RP). The aim of this study is to differentiate between CIP and RP by pretreatment CT radiomics and clinical or radiological parameters.

**Methods:**

A total of 126 advance-stage NSCLC patients with pneumonitis were enrolled in this retrospective study and divided into the training dataset (*n* =88) and the validation dataset (*n* = 38). A total of 837 radiomics features were extracted from regions of interest based on the lung parenchyma window of CT images. A radiomics signature was constructed on the basis of the predictive features by the least absolute shrinkage and selection operator. A logistic regression was applied to develop a radiomics nomogram. Receiver operating characteristics curve and area under the curve (AUC) were applied to evaluate the performance of pneumonitis etiology identification.

**Results:**

There was no significant difference between the training and the validation datasets for any clinicopathological parameters in this study. The radiomics signature, named Rad-score, consisting of 11 selected radiomics features, has potential ability to differentiate between CIP and RP with the empirical and α-binormal-based AUCs of 0.891 and 0.896. These results were verified in the validation dataset with AUC = 0.901 and 0.874, respectively. The clinical and radiological parameters of bilateral changes (*p* < 0.001) and sharp border (*p* = 0.001) were associated with the identification of CIP and RP. The nomogram model showed good performance on discrimination in the training dataset (AUC = 0.953 and 0.950) and in the validation dataset (AUC = 0.947 and 0.936).

**Conclusions:**

CT-based radiomics features have potential values for differentiating between patients with CIP and patients with RP. The addition of bilateral changes and sharp border produced superior model performance on classifying, which could be a useful method to improve related clinical decision-making.

## Introduction

Immune checkpoint inhibitors (ICIs) have established a new paradigm for cancer therapeutic and created many breakthroughs in clinical practice. Consolidation immunotherapy of immune checkpoint inhibitors following concurrent chemoradiotherapy (CCRT) is the current standard of care for patients with unresectable locally advance-stage non-small cell lung cancer (NSCLC) (1, 2). While the combination of ICIs and radiotherapy (RT) has shown promising prospects, treatment-related pneumonitis, including checkpoint inhibitor-related pneumonitis (CIP) and radiation pneumonitis (RP) (3, 4), one of the most frequent and clinically challenging adverse events in the combination setting, should raise concerns.

CIP is a rare but seriously adverse event with incidence of approximately 5% in any grade and <3% in grade 3 or higher level for NSCLC patients who received ICIs (5). Meanwhile, approximately 60% of patients with thoracic tumors receive RT at some point during the course of the disease (6). RT contributed to lung injury and induced pneumonitis. The incidence of radiation pneumonitis ranges from 4 to 10% in grade 3 or higher in lung cancer patients after radiotherapy within 6 months (7). Differentiating a diagnosis between CIP and RP is a difficult challenge in clinical practice. The clinical symptoms of CIP seem to be non-specific as far as clinical manifestations are being concerned. Clinical symptoms, such as fever, cough, and shortness of breath, are found in both CIP and RP (8). Additionally, the thoracic CT radiological findings of CIP are similar to the RP model, especially in the early stage of the disease course. Although some studies have reported the typical radiological findings of CIP and RP, these manifestations are only suggestive due to pneumonitis having a wide range of radiological appearance (9). Furthermore, these patients are at risk for both ICI- and RT-induced pneumonitis, and a differentiating diagnosis can have an important effect on clinical management, such as the decision to continue or restart the ICI treatment.

Radiomics is inspired by the combination of artificial intelligence and medical imaging. High-throughput and quantitative images of features reflect the underlying pathophysiology and reveal information on pathogenesis and etiology (10). CT radiomics analysis, as an interdisciplinary technique, has been widely used in distinguishing between benign and malignant tumors (11), predicting the prognosis of patients with a tumor (12), monitoring therapeutic responses (13), and gene expression (14), yet scanty attention has been paid to investigating how to distinguish between CIP and RP using radiomics features for patients with NSCLC. Given the previous conclusions, whether CT radiomics can be used for the identification of CIP and RP becomes crucial and worth exploring. Theoretically, the characteristics of pneumonitis provided by CT hide the potential correlation with pneumonitis etiology and can be quantitatively analyzed. It is expected that the high-throughput and high-dimensional CT radiomics features play a vital role in distinguishing between CIP and RP. We hypothesized that constructing a model and developing a quantitative tool could improve the diagnostic efficiency for CIP and RP *via* analysis of CT radiomics features and clinical or radiological parameters.

## Materials and Methods

### Study Design and Workflow

Firstly, NSCLC patients who were treated with ICIs or RT and developed pneumonitis were enrolled in this study. Chest CT images were collected for subsequent radiomics analysis. After that, radiomics features were extracted from regions of interest (ROIs). Then, radiomics features were selected based on their effectiveness in differentiating the subtype of pneumonitis. Next, Rad-scores and nomogram were constructed and evaluated in the training and validation datasets.

### Patient Selection

In this study, a total of 126 NSCLC patients who received radiotherapy or immune checkpoint inhibitors and developed pneumonitis between April 2018 and August 2021 at Shandong Provincial Hospital Affiliated to Shandong First Medical University were recruited. The inclusion criteria were as follows: (1) pathological diagnosis of NSCLC by biopsy or bronchofiberoscopy, (2) standard chest CT scans, and (3) collection of CT images and clinical or radiological parameters of patients who developed pneumonitis. The exclusion criteria were as follows: (1) insufficient image quality, such as artifacts, (2) with treatment history of thoracic surgery, and (3) with the history of anti-tumor or anti-inflammatory therapy. In this study, pneumonitis was defined as immune checkpoint inhibitor-related and radiation pneumonitis which did not occur owing to other confirmed reasons such as bacterial and virus infections. The subtype of pneumonitis was determined by the following procedures. Patients with CIP were identified by a history of using immune checkpoint inhibitors, nonproductive cough, fever, and other clinical symptoms. Varied radiographic findings, such as from chest CT imaging, should be considered, such as cryptogenic organizing pneumonia, pneumonitis presenting as acute interstitial pneumonia, acute respiratory distress syndrome, *etc.* The RP datasets that consisted of 69 patients were randomly enrolled from NSCLC patients who were treated with thoracic radiotherapy and developed RP within 6 months after RT. The symptoms include shortness of breath, nonproductive cough, fever, and other clinical symptoms. The definitions of CIP and RP were consistent with the previous guidelines (15, 16). Clinical factors, including gender, age, smoking history, histology, and radiological findings—such as number of lobes, volume of lung, bilateral changes, and sharp border—were recorded.

### CT Image Acquisition

CT images were collected from all enrolled patients. All CT scans were acquired from a 128-row CT scanner (Philips iCT 128, Philips Medical System, The Netherlands). The CT scans were acquired with the following protocols: tube voltage, 120 kV; tube current, ranging from 300 to 400 mA; slice thickness, 3 mm; matrix size, 512 × 512; in-plane resolution, 0.8142 × 0.8142 mm^2^; and helical scanning mode.

### Region of Interest

**Figure 1:**
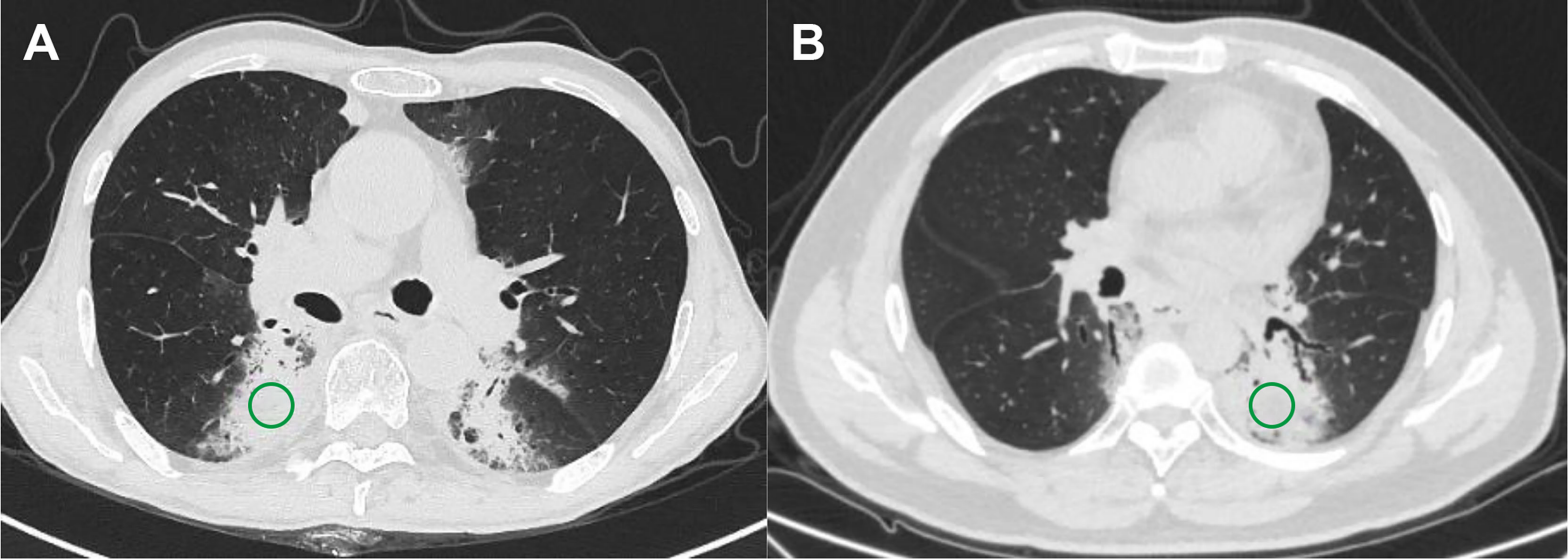
Examples of regions of interest for two subtypes of pneumonitis. **(A)** Checkpoint inhibitor-related pneumonitis on routine CT and **(B)** radiation pneumonitis on routine CT.

The ROIs, defined as the lung injury region visualized on the lung parenchyma window of CT images, were delineated by two experienced radiologist and oncologist in all CT scans using AccuContour software (version 3.0, Manteia Medical Technologies Co. Ltd., Xiamen, China). Given that a larger variability existed in the border of the pneumonitis region, cylindrical ROIs of diameter 20 mm and height 15 mm (consecutive five slices) were contoured to ensure that the features are valuable and correct. Then, the contoured ROIs were checked and modified slice by slice by another experienced radiologist. Examples of contoured ROIs on the lung parenchyma window of CT images are depicted in **Figure 1**.

### Feature Extraction

Radiomics features were extracted using embedded radiomics computational module-based PyRadiomics packages that enable feature calculation in the 3D slicer (version 4.11.2, www.slicer.org) software. In this study, 93 radiomics features were extracted from original CT images, including (1) 18 first-order intensity histogram (IH)-based features and statistical matrix (SM)-based features divided into (2) 24 gray-level co-occurrence matrix-based features, (3) 16 gray-level run-length matrix-based features, (4) 16 gray-level size zone matrix-based features, (5) 5 neighboring gray-tone difference matrices, and (6) 14 gray-level dependence matrix features. Moreover, 744 wavelet-based features (including IH and SM features) were extracted from eight wavelet decompositions.

### Radiomics Signature and Nomogram Construction

Before radiomics signature building, feature selection was implemented to keep the signature more robust and effective. In this study, least absolute shrinkage and selection operator (LASSO) (with a binary regression model, a five-fold cross-validation method, an “auc” loss measurement, and using non-normalized data) was performed to determine the most predictive features. After feature selection, a radiomics signature, also termed Rad-score, was established from a linear combination of selected features and corresponding coefficients derived from LASSO.

Moreover, to explore whether clinical or radiological parameters will add more benefit for differentiating subtypes of pneumonitis, nomograms were constructed by incorporating Rad-score and clinical factors compared to Rad-score alone. Notably, the clinical factors used for nomogram establishment were tested *via* univariate analysis.

### Validation of Radiomics Signature and Nomogram

The correlation between the Rad-score and subtype of pneumonitis was evaluated using the receiver operating characteristic curve (ROC) and area under the curve (AUC). Due to the limited sample size of patients, inevitable bias exists in the uneven appearance of the empirical ROC, which will result in a lower or higher estimated performance. Therefore, ROC curves may perform poorly for evaluation despite a superior AUC when the positive and negative data used for building a prediction model are imbalanced. Therefore, a precision–recall curve (PRC) that plots the positive prediction value against the true positive rate across all thresholds was recommendable for such case. It represents a more accurate method to assess established classification models, and the area under PRC is defined as average precision (AP). Consequently, the α-binomial model-based ROC curve and PRC proposed by Brodersen et al. to plot smooth curves were used to address the above-mentioned issues in this study (17). The discrimination of nomogram was evaluated by α-binomial model-based ROC curve and PRC. Meanwhile, the agreement between the actual CIP probability and predicted CIP probability was assessed by a calibration curve, and Hosmer–Lemeshow test was utilized to determine the agreement; a *p*-value >0.05 indicates good agreement. Finally, the Rad-score and the nomogram were compared using decision curve analysis with regards to clinical utility.

### Statistical Analysis

Statistical analyses were performed in R software (version 3.3.1). Comparisons, calibration curve, decision curve analysis, and univariate analyses were implemented in R with the “stats” and “rms” packages. Chi-square test or Fisher’s test was used to analyze the categorical variables. Mann–Whitney *U*-tests were employed to compare the patients’ continuous characteristics where appropriate. In this study, univariate analysis was performed by Spearman’s correlation test, and a coefficient higher than 0.85 indicates that the clinical factors are correlated to the subtype of pneumonitis. LASSO was performed in R with the *glmnet* package. The reported statistical significance levels were all two-sided. The statistical significance level was set to 0.05.

## Results

### Patient Characteristics

In total, 126 consecutive patients were enrolled in this study and were divided into training and validation datasets with a ratio of 7:3, including 88 and 38 patients, respectively. The clinical and radiological factors are summarized in **Table 1**. There were no significant differences between these factors in the two sets, including gender, age, and radiological findings.

**Table 1:** Clinical and radiological parameters of patients with pneumonitis in the training and validation datasets.

Characteristic	Training dataset	Validation dataset	*p*
Gender			0.502
Male	47	21	
Female	41	17	
Age (years)			0.424
Mean	57.1	59.4	
Smoking history			0.351
Yes	58	23	
No	30	15	
Number of lobes			0.236
≤3	54	20	
>3	34	18	
Volume of lung			0.310
<10%	21	14	
10%≤ *X <*50%	42	16	
≥50%	25	8	
Histology			0.547
Adenocarcinoma	61	26	
Squamous cell carcinoma	27	12	
Radiological elements			0.398
Ground-glass opacities (GGO)	33	12	
Consolidation	16	11	
GGO + consolidation	39	15	
Bilateral changes			0.498
Yes	40	18	
No	48	20	
Sharp border			0.350
Yes	42	16	
No	46	22	
Subtype of pneumonitis			0.549
Checkpoint inhibitor-related pneumonitis	40	17	
Radiation pneumonitis	48	21	

### Radiomics Signature Construction and Validation

**Figure 2:**
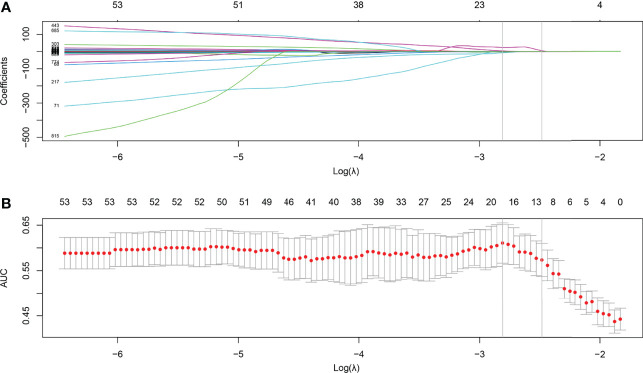
Feature selection using the least absolute shrinkage and selection operator (LASSO) with a binary regression model. **(A)** The LASSO coefficient profile was plotted using coefficients against log(*λ*). **(B)** Tuning parameter against parameter log(*λ*). The areas under the curve were depicted with corresponding *λ*. The vertical lines are maximum and 1-standard error (1-se) criteria, respectively. As a result, 12 radiomics features with nonzero coefficients were selected using 1-se criteria.

After feature selection using LASSO binary regression model, 11 radiomics features remained with non-zero coefficients, and the results are illustrated in **Figure 2**. **A** Rad-score was established using a linear combination of selected predictive features and corresponding coefficients. The formula for calculating the Rad score is as follows:


Rad−score=wavelet.LLH.ngtdm.Coarseness×4.901552+wavelet.LHL.glcm.ClusterShade×0.001658+wavelet.LHL.glrlm.LRHGLE×0.00095+wavelet.LHL.glszm.ZoneVariance×1.87E−06+wavelet.LHH.firstorder.Median×0.104804−wavelet.HLL.firstorder.Kurtosis×0.03584+wavelet.HLL.firstorder.Skewness×0.53657−wavelet.HHL.firstorder.Mean×1.27905−wavelet.HHL.firstorder.Skewness×0.73588−wavelet.HHH.gldm.DependenceEntropy×0.20179+wavelet.LLL.firstorder.Minimum×1.03E−05

**Figure 3:**
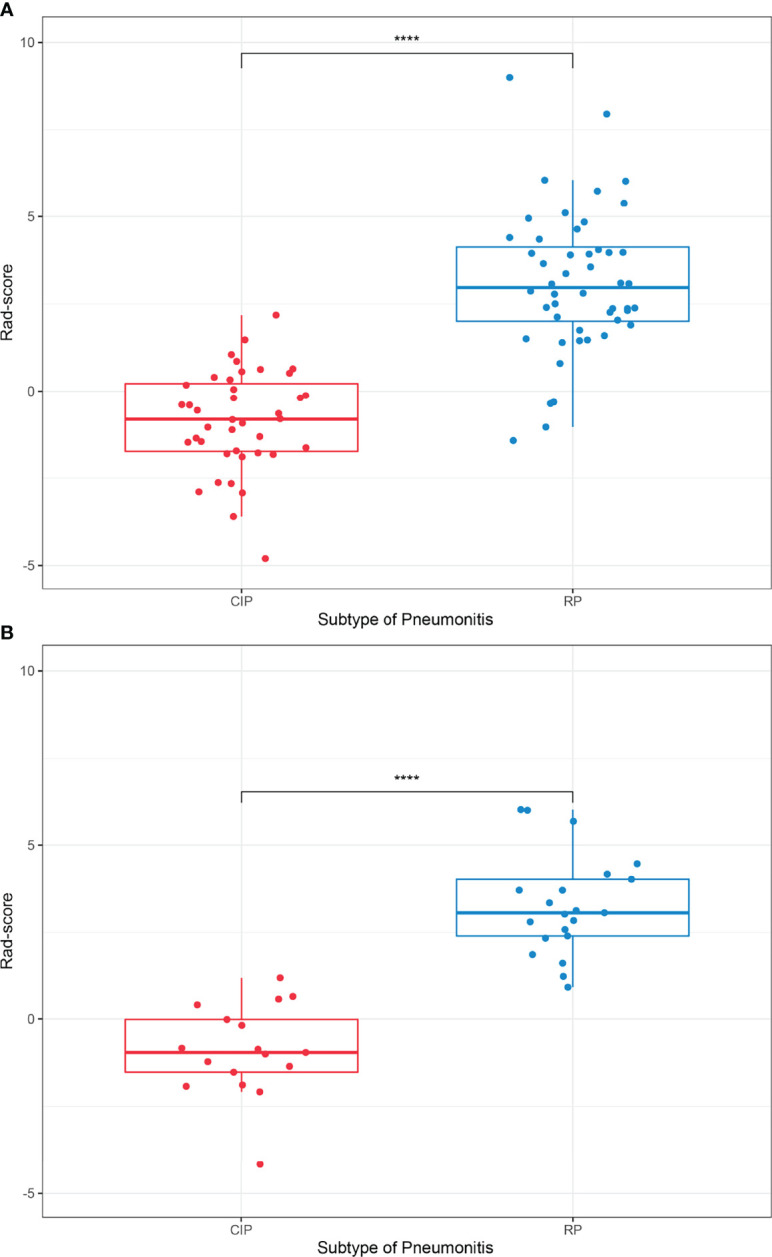
Boxplots for subtypes of lung injury in the **(A)** training and **(B)** validation datasets, respectively. ****p-value <0.0001.

**Figure 4:**
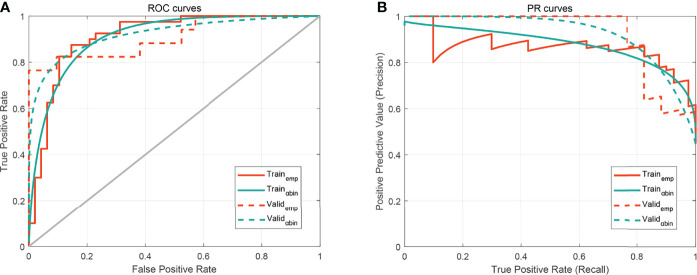
The performance of the developed Rad-score. **(A)** Receiver operating characteristic (ROC) curves. **(B)** Precision–recall curve (PRC). The subscripts emp and αbin mean empirical and α-binormal-based ROC or PRC, respectively.

We plotted box plots to show the statistical distribution of Rad-scores in the training and validation datasets, as shown in **Figure 3**. There were statistically significant differences between the training and validation datasets (all *p*-values <0.0001). The empirical and α-binormal-based ROC curves and PRCs of the established Rad-score are displayed in **Figure 4**. The empirical and α-binormal AUCs and APs of Rad-score are summarized in **Table 2**. The Rad-score achieved a good performance for making a distinction between CIP and RP in NSCLC patients with AUC_αbin_ = 0.891 (95%CI, 0.876–0.906) and AUC_emp_ = 0.896 (95%CI, 0.879–0.913), respectively. Similar results were committed in the validation dataset; the above-mentioned Rad-score showed a favorable assessment efficacy with AUC_αbin_ = 0.901 (95%CI, 0.855–0.947) and AUC_emp_ = 0.874 (95%CI, 0.843–0.905) for discriminating between patients with CIP and RP. The results of Rad-score had achieved a satisfactory performance.

**Table 2:** Comparison of the performances of Rad-score and nomogram.

Performances	Training cohort				Validation cohort			
	AUC_αbin_	AUC_emp_	AP_αbin_	AP_emp_	AUC_αbin_	AUC_emp_	AP_αbin_	AP_emp_
Rad-score	0.891	0.896	0.857	0.811	0.901	0.874	0.903	0.891
	(0.876–0.906)	(0.879–0.913)	(0.836–0.878)	(0.785–0.837)	(0.855–0.947)	(0.843–0.905)	(0.860–0.946)	(0.859–0.923)
Nomogram	0.953	0.950	0.949	0.926	0.947	0.936	0.943	0.887
	(0.916–0.990)	(0.941–0.959)	(0.935–0.963)	(0.900–0.952)	(0.912–0.982)	(0.905–0.967)	(0.925–0.961)	(0.860–0.914)
*P*-value	<0.001ζ	<0.001ϵ	<0.001η	<0.001θ	<0.001ψ	<0.001σ	<0.001ρ	<0.001μ

All the data in parentheses are 95% confidence interval (CI) values. The subscripts emp and αbin means empirical-based and α-binormal-based area under the curve (AUC) or average precision (AP), respectively. ζ, ψ—the comparison of AUCαbin; ϵ, σ—the comparison of AUCemp; η, ρ—the comparison of Apαbin; and θ, μ—the comparison of APemp between Rad-score and nomogram in both training and validation cohorts.

### Nomogram Construction and Validation

Before nomogram construction, univariate analyses were performed for clinical factors and radiological features using chi-square test or Mann–Whitney *U*-tests. In the training dataset, the differences of clinicopathological characteristics and radiological findings between the patients with CIP and RP are shown in **Table 3**. The median age of patients with CIP and RP were 56.8 and 57.4, and there was no discrepancy in the subtype of pneumonitis by gender (*p* = 0.313) and smoking history (*p* = 0.200). Moreover, there was a trend towards more lung lobes (*p* = 0.184) and volumes (*p* = 0.101) infected in patients with radiation pneumonitis compared with patients with CIP. We did not figure out the values of histology (*p* = 0.067), radiological elements (*p* = 0.910), and other factors for classification between CIP and RP. The variables of bilateral changes (*p* < 0.001) and sharp border (*p* = 0.001) were considered independent biomarkers and had a statistically significant difference between CIP and RP.

**Table 3:** Clinical and radiological parameters of patients with checkpoint inhibitor-related pneumonitis (CIP) and radiation pneumonitis (RP) in the training dataset.

Characteristic	CIP	RP	*p*
Gender			0.313
Male	23	24	
Female	17	24	
Age (years)			0.481
Mean	56.8	57.4	
Smoking			0.200
Yes	24	34	
No	16	14	
Number of lobes			0.184
≤3	22	32	
>3	18	16	
Volume of lung			0.101
<10%	10	11	
10%≤ *X <*50%	23	19	
≥50%	7	18	
Histology			0.067
Adenocarcinoma	24	37	
Squamous cell carcinoma	16	11	
Radiological elements			0.910
Ground-glass opacities (GGO)	15	18	
Consolidation	8	8	
GGO + consolidation	17	22	
Bilateral changes			<0.001
Yes	27	13	
No	13	35	
Sharp border			0.001
Yes	11	31	
No	29	17	

**Figure 5:**
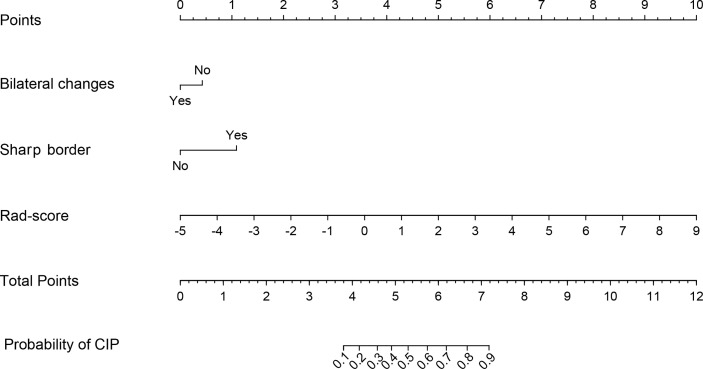
Nomograms constructed in this study using the training dataset.

**Figure 6:**
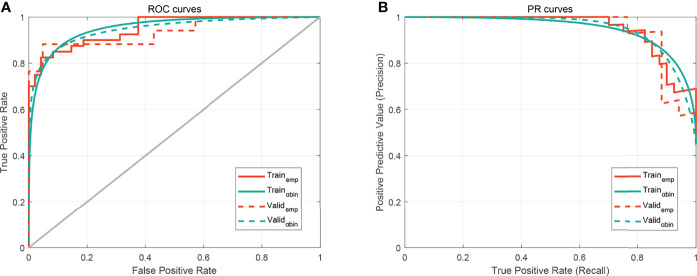
The performance of the developed nomogram. **(A)** Receiver operating characteristic (ROC) curves. **(B)** Precision–recall curve (PRC). The subscripts emp and αbin mean empirical and α-binormal-based ROC or PRC, respectively.

To determine the benefits for prediction performances of radiomics features, the Rad-score, bilateral changes, and sharp border were incorporated into the radiomics nomogram, as shown in **Figure 5**. In **Figure 6**, the nomogram model displayed the highest discrepancy between CIP and RP with the empirical and α-binormal-based AUC of 0.953 and 0.950 in the training set. In the validation samples, the nomogram yielded the greatest AUC (0.947, 95% CI: 0.912–0.982; 0.936, 95%CI: 0.905–0.967), which confirmed that the nomogram model achieved better differential capacity than the Rad-score. As illustrated by the results, an obvious separation between CIP and RP was detected in the validation dataset with AP of 0.943 and 0.887 (as shown in **Table 2**).

**Figure 7:**
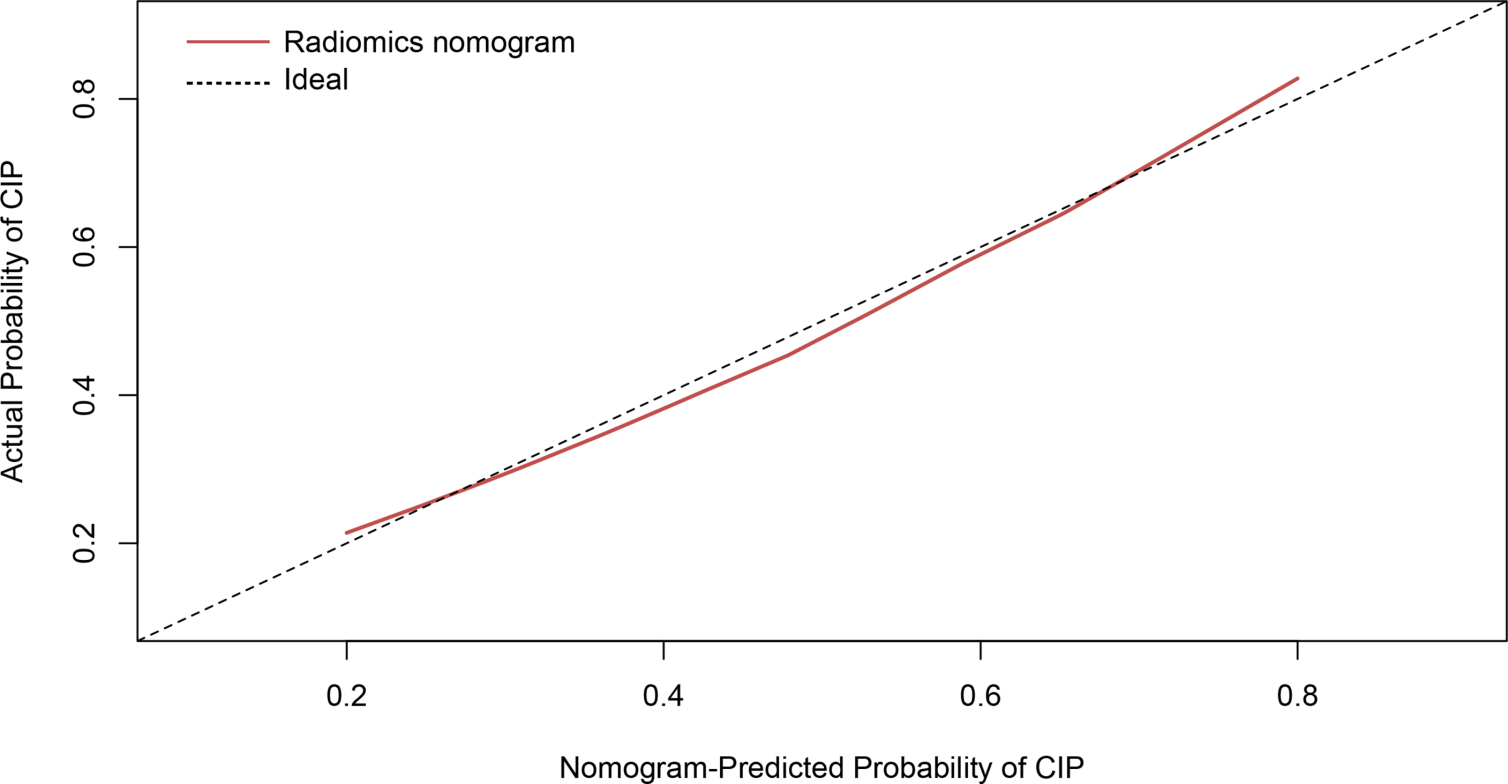
Calibration curve of the radiomics nomogram presented as a solid red line. The diagonal dashed line indicates perfect agreement.

**Figure 8:**
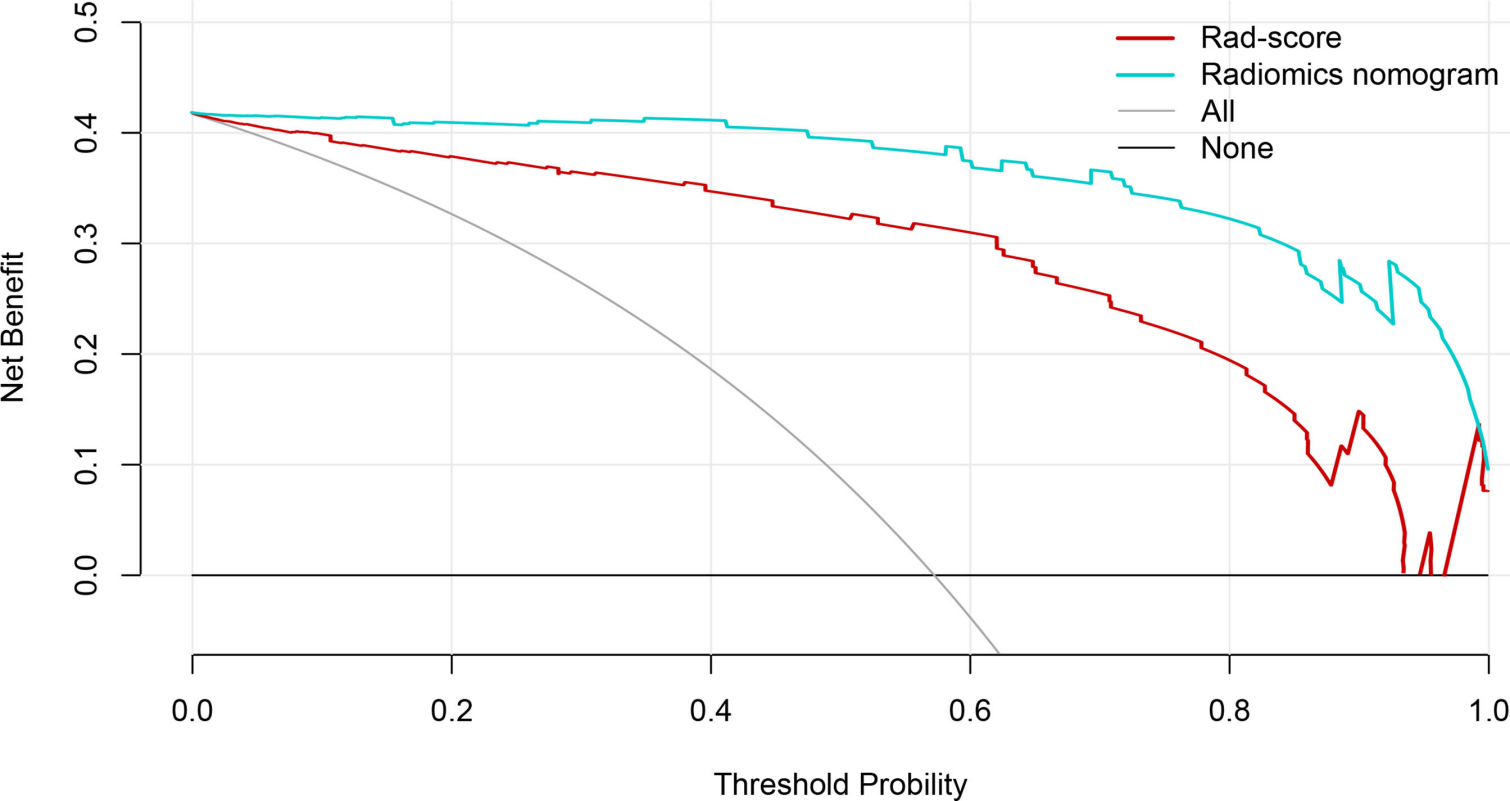
The decision curves of the Rad-score, radiomics nomogram, and two extreme curves were plotted based on the validation dataset. It showed that the use of the radiomics nomogram to predict CIP probability in patients with non-small cell lung cancer has a greater benefit than the use of Rad-score.

The calibration curve manifested sufficient consistency between estimated CIP probability using the nomogram and the actual observed outcome in **Figure 7**. The closer the calibration curve was to the diagonal, the better the predictive ability of the nomogram. The *P*-value of Hosmer–Lemeshow test for subtype of pneumonitis was 0.511. To determine the clinical utility, decision curves were plotted for Rad-score and radiomics nomogram (shown in **Figure 8**). It showed that the radiomics nomogram produced a greater net benefit than the Rad-score.

## Discussion

With the evolution of immunotherapy, the combination of immunotherapy and radiotherapy became increasingly important in guiding NSCLC treatment according to the latest NCCN guidelines (18). However, the widespread utilization of ICIs has contributed in off-target immune-related adverse events, especially CIP. In this retrospective study, we developed and validated a classification model based on CT radiomics for distinguishing between CIP and RP. Radiomics nomograms were extracted and showed good performance on the training and validation datasets with AUC = 0.953 and 0.947, which proved that the CT-based radiomics nomogram was feasible. In the radiomics nomogram, Rad-score has the largest weight and is more important for distinguishing subtypes of pneumonitis. All features used to construct the Rad-score were wavelets; the reasonable hypothesis was that the similar pixel intensity of different pneumonitis derived from original images provide limited differentiation power in the modeling process. However, multiple frequency decomposition of the original CT image can decode the hidden difference of phenotype between CIP and RP (19). As far as we are concerned, this is the first study that uses comprehensive information by including both radiomics, clinical, and radiological factors in the classification of CIP and RP.

Previous studies demonstrated the safety of thoracic RT in patients receiving ICIs (20). Nevertheless, current evidence has suggested that radiotherapy had immune stimulating effects, which could potentially enhance the effectiveness of ICIs and increase the risk of relevant immune-related adverse events (21). Preliminary studies mentioned the radiologic appearances and clinical symptoms of CIP, which were similar with RP characteristics (22). The clinical symptoms of pneumonitis after immune checkpoint inhibitors or radiotherapy included cough, dyspnea, fever, and shortness of breath. Moreover, the most frequent CT image findings are ground-glass opacities or consolidative opacities in subpleural regions among patients with CIP (23). Generally, distinguishing between CIP and RP has a crucial impact on the treatment to pneumonitis and decision-making on therapy in the near feature.

Since the occurrence of CIP, CT morphological features have been treated as important clues for diagnosing CIP. Our present study revealed that these features were valuable but not the only strong clue for diagnosing CIP or RP. We noted that bilateral changes and a sharp border showed significant difference for identifying CIP and RP. ICI pneumonitis trended toward a higher likelihood of bilateral CT changes (*p* < 0.001) than pneumonitis from RT alone. Patients with CIP were less likely to have a sharp border (*p* = 0.001) in comparison with RP. This conclusion was consistent with previous studies (24). On the other hand, the Rad-score and nomogram were evaluated by α-binominal model-based ROC and PRCs and compared to empirical curves. In this study, the results showed that no significant difference was observed between the two types of curve. Besides this, performance measures based on PRCs were helpful to supplement and validate the ROC curves. It can effectively address the practical limitations derived from empirical curves (17). Furthermore, the decision curves demonstrated that radiomics nomogram added more benefits for differentiating CIP from RP than Rad-score. Generally, the nomogram was robust and accurate for distinguishing the subtype of pneumonitis.

Our result verified the values of quantitative radiomics features, instead of qualitative radiological factors, to discriminate pneumonitis etiology. Recently, a study performed a radiomics analysis and extracted 1,860 features in a total of 290 patients who were treated with immunotherapy (25). Radiomics features were identified and predicted subsequent immunotherapy-related pneumonitis. In accordance with RP, some researchers stated that the multi-region CT radiomics features can help to predict grade ≥2 RP for NSCLC patients (26). As Du et al. proposed, a radiomics model based on thoracic cone-beam computed tomography had a potential value of RP prediction (27). The radiomics features had an ability to assess the heterogeneity of image intensities and highlight small differences that cannot be recognized by the naked eyes. Yang et al. utilized CT-based radiomics to differentiate COVID-19 from other pneumonias in patients with accuracy of 89.83% and AUC of 0.940 (28). Tabatabaei et al. inferred that radiomics features, conjoined with random forest and neural network, appeared to yield promising results in distinguishing between COVID-19 and H1N1 influenza by CT scanning, with AUC = 0.970 (29).

As far as we know, few studies focused on this topic. Chen et al. extracted radiomics features from CT images and only trained 29 patients with RT and 23 patients with ICIs (30). The classifier has shown a performance on the training dataset with AUC =0.79 and the validation dataset with AUC = 0.840. The results were in line with ours, but the predictive efficacy of this model was much lower. Cheng et al. collected the CT images of 73 NSCLC patients with ICI or RT and extracted only 3 types of radiomics features (31). They figured out that the kind of bag-of-word features achieved an AUC of 0.937. Despite the encouraging results, there were some limitations in the above-mentioned study. Firstly, the small sample size and limited radiomics features that resulted in this conclusion can be hardly generalized. Secondly, no classification model, such as radiomics signatures or nomogram, has been built for discriminating between CIP and RP. Our results indicated that an integrated model combined with radiomics features and clinical factors may help to differ CIP from RP in NSCLC. Thirdly, there were no clinical and laboratory parameters involved, which have been proved to reveal the mechanism of two kinds of pneumonitis to some extent (32, 33).

This difference in the pathogenesis of pneumonitis remains to be discussed. To our best knowledge, Chen et al. mentioned that checkpoint inhibitor pneumonitis was more likely to involve more lobes of the lung (24). Based on our wide clinical experience and practice, RP was commonly limited in the radiation field of the lung, while CIP was always irregularly distributed in random fields of the lung. Moreover, lymphocytes were predominant to the pathogenesis of pneumonitis. The relationship between neutrophil-to-lymphocyte ratio and CIP may provide a reasonable explanation (34). The value of inflammatory cytokine in the diagnosis of CIP has not yet been completely evaluated. IL-10, an anti-inflammatory cytokine, was maintained at a lower level in RP patients at baseline (35). In contrast, lung cancer patients with CIP, shown to have increased IL-6, were associated with the occurrence of disease (36).

It is necessary to identify the mechanism of pneumonitis arising from immunotherapy and radiotherapy. Radiotherapy could directly damage DNA and reactive oxygen species generation, then mediate intracellular signaling, and result in the release of molecules and cytokines (37). The occurrence of acute pneumonitis and chronic pulmonary fibrosis is mainly through the TGF-β/Smad (38), HMGB1/TLR4 (39), and Nrf2/ARE signaling pathways (40). Glucocorticoid drugs are the mainstay of RILI treatment in clinical practices. Regarding IRLI, generalized immune activation owing to checkpoint neutralization, preexisting autoantibodies, and off-target effects of T cell-mediated immunity will lead to immune-related lung injury (41). Immunosuppressive drugs such as glucocorticoids, mycophenolate mofetil, and cytokine inhibitors were mainly used for IRLI treatment (42). Zhang et al. reported the crosstalk among signaling pathways in RILI and IRLI, which noted that the TGF-β signaling pathway played an important role in the crosstalk between IRIL and RILI (41); more exploration and discovery are required to pinpoint the mechanisms of RILI and IRLI.

There are some limitations in this study. First, it is a retrospective study with 126 patients. A larger sample size and prospective study are necessary to explore and validate in the near future. Second, training and validation datasets were acquired from a single institution, which resulted in a conclusion hardly generalizable to other study centers. Further investigation will concentrate on samples from various institutions as external validation dataset. Third, no other laboratory parameters were included in the analysis. We trust that the subsequent research should be to develop a robust model.

## Conclusions

In summary, there are many similarities of clinical symptoms and radiological findings between immune checkpoint inhibitor-related pneumonitis and radiation pneumonitis for patients with NSCLC, which propose great management challenges in clinical practice. Our results have successfully suggested that CT radiomics features are capable of differentiating between CIP and RP. Additions of bilateral changes and sharp border produce a superior nomogram model performance. Our findings suggest that a radiomics nomogram could be used for clinical decision-making and providing an accurate diagnostic tool.

## Data Availability Statement

The original contributions presented in the study are included in the article/supplementary material. Further inquiries can be directed to the corresponding author.

## Ethics Statement

Written informed consent was obtained from the individual(s) for the publication of any potentially identifiable images or data included in this article.

## Author Contributions

QQ designed the study and wrote the manuscript. QW participated in the study designing and data collection. YW and AF provided the analysis of data and ROI segmentation. QQ and QW participated in data collection and offered guidance. LX carried out the study design and interpretation of data and drafted the manuscript. All authors contributed to the article and approved the submitted version.

## Funding

This study was supported by the National Natural Science Foundation of China (grant number 82001902), Shandong Provincial Natural Science Foundation (grant numbers ZR2020QH198 and ZR2020QH200), Radiation Oncology Translational Medicine Foundation for Scientific Research of Bethune (grant number flzh202123), and the Special Tumor Foundation for Scientific Research of Saifu (grant number fszl202106). The funding sources had no role in the study design, data collection, analysis of interpretation, or writing of this manuscript.

## Conflict of Interest

The authors declare that the research was conducted in the absence of any commercial or financial relationships that could be construed as a potential conflict of interest.

## Publisher’s Note

All claims expressed in this article are solely those of the authors and do not necessarily represent those of their affiliated organizations, or those of the publisher, the editors and the reviewers. Any product that may be evaluated in this article, or claim that may be made by its manufacturer, is not guaranteed or endorsed by the publisher.
